# The (*G*>*A*) rs11573191 Polymorphism of *PLA2G5* Gene Is Associated with Premature Coronary Artery Disease in the Mexican Mestizo Population: The Genetics of Atherosclerotic Disease Mexican Study

**DOI:** 10.1155/2014/931361

**Published:** 2014-05-18

**Authors:** Gilberto Vargas-Alarcón, Carlos Posadas-Romero, Teresa Villarreal-Molina, Edith Alvarez-León, Javier Angeles-Martinez, María Elena Soto, Irma Monroy-Muñoz, Juan Gabriel Juárez, Carlos Jerges Sánchez-Ramírez, Julian Ramirez-Bello, Silvestre Ramírez-Fuentes, José Manuel Fragoso, José Manuel Rodríguez-Pérez

**Affiliations:** ^1^Departments of Molecular Biology, Endocrinology, and Immunology, National Institute of Cardiology Ignacio Chávez, 14080 Mexico City, DF, Mexico; ^2^Department of Endocrinology, National Institute of Cardiology Ignacio Chávez, Juan Badiano 1, Sección XVI, Tlalpan, 14080 Mexico City, DF, Mexico; ^3^Cardiovascular Genomics Laboratory, National Institute of Genomic Medicine, 14610 Mexico City, DF, Mexico; ^4^Department of Immunology, National Institute of Cardiology Ignacio Chávez, Juan Badiano 1, Sección XVI, Tlalpan, 14080 Mexico City, DF, Mexico; ^5^Laboratory of Genomic Medicine, Research Unit, Juárez de México Hospital, 07760 Mexico City, DF, Mexico

## Abstract

Coronary artery disease (CAD) is a multifactorial disorder that results from an excessive inflammatory response. Secretory phospholipase A_2_-V (sPLA_2_-V) encoded by *PLA2G5* gene promotes diverse proinflammatory processes. The aim of the present study was to analyze if *PLA2G5* gene polymorphisms are associated with premature CAD. Three *PLA2G5* polymorphisms (rs11573187, rs2148911, and rs11573191) were analyzed in 707 patients with premature CAD and 749 healthy controls. Haplotypes were constructed after linkage disequilibrium analysis. Under dominant, recessive, and additive models, the rs11573191 polymorphism was associated with increased risk of premature CAD (OR = 1.51, *P*_dom_ = 3.5 × 10^−3^; OR = 2.95, *P*_rec_ = 0.023; OR = 1.51, *P*_add_ = 1.2 × 10^−3^). According to the informatics software, this polymorphism had a functional effect modifying the affinity of the sequence by the MZF1 transcription factor. *PLA2G5* polymorphisms were in linkage disequilibrium and the *CGA* haplotype was associated with increased risk of premature CAD (OR = 1.49, *P* = 0.0023) and with hypertension in these patients (OR = 1.75, *P* = 0.0072). Our results demonstrate the association of the *PLA2G5* rs11573191 polymorphism with premature CAD. In our study, it was possible to distinguish one haplotype associated with increased risk of premature CAD and hypertension.

## 1. Introduction

Coronary artery disease (CAD) is a complex multifactorial and polygenic disorder resulting from an excessive inflammatory response to various forms of injurious stimuli to the arterial wall [1–3]. Although the precise mechanisms responsible for the onset of the disease are still unknown, multiple genetic factors may cooperate with environmental factors to confer susceptibility to CAD. The secretory phospholipase A_2_ (sPLA_2_) family of enzymes hydrolyzes the sn-2 ester bond of phospholipids and cell membranes, generating nonesterified free fatty acids and lysophospholipids, which may promote diverse proinflammatory processes [4].

Ten sPLA_2_ enzymes have been described in humans and four of them (sPLA_2_-IIA, sPLA_2_-III, sPLA_2_-V, and sPLA_2_-X) have been implicated in atherosclerosis [5–11]. Hydrolysis by sPLA_2_-V reduces the capacity of HDL to promote cellular cholesterol efflux from lipid-loaded macrophages [12]. Some experiments have shown that LDL hydrolyzed by sPLA_2_-V induces foam cell formation in mouse peritoneal macrophages [6, 7]. On the other hand, immunohistochemical analysis has shown sPLA_2_-V to be associated with smooth muscle cells and foam cells in the lipid cores of both human and mouse atherosclerotic lesions [13]. The sPLA_2_-V is encoded by the* PLA2G5* gene located in chromosome 1p34-36.1 [14]. Polymorphisms in this gene have been reported and some of them have been associated with LDL and oxLDL levels in a group of patients with type II diabetes mellitus [15]. These data suggest that the gene that encodes sPLA_2_-V could be an important candidate gene to be studied in atherosclerosis. The aim of the present study was to analyze if* PLA2G5* gene polymorphisms are associated with premature coronary artery disease (CAD) in a case-control association study (GEA or genetics of atherosclerotic disease).

## 2. Material and Methods

The primary aim of the GEA study is to investigate genetic factors associated with premature CAD and other coronary risk factors in the Mexican population. The study complies with the Declaration of Helsinki. All participants provided written informed consent, and the study was approved by the Ethics Committees of the Instituto Nacional de Cardiología ‘‘Ignacio Chávez” and the Instituto Nacional de Medicina Genómica.

### 2.1. Subjects

All GEA participants are unrelated and of self-reported Mexican-Mestizo ancestry (three generations). A Mexican Mestizo is defined as someone born in Mexico, who is a descendant of the original autochthonous inhabitants of the region and of individuals, mainly Spaniards, of Caucasian and/or African origin, who came to America during the sixteenth century. The study included 707 patients with premature CAD and 749 healthy controls from the genetics of atherosclerotic disease (GEA) Mexican study. The selection of patients and controls of the GEA study has been described previously [16]. Demographic, clinical, anthropometric, and biochemical parameters, as well as cardiovascular risk factors, were evaluated in patients and controls.

### 2.2. Genetic Analysis

Genomic DNA from whole blood containing EDTA was isolated by standard techniques. The rs11573185, rs2148911, and rs11573191 single nucleotide polymorphisms (SNPs) of the* PLA2G5* were genotyped using 5′ exonuclease TaqMan genotyping assays on an ABI Prism 7900HT Fast Real-Time PCR system, according to manufacturer's instructions (Applied Biosystems, Foster City, CA, USA).

### 2.3. Statistical Analysis

All calculations were performed using SPSS version 18.0 (SPSS, Chicago, IL) statistical package. Means ± SD and frequencies of baseline characteristics were calculated. Chi-square tests were used to compare frequencies and ANOVA and Student's* t*-test were used to compare means. ANCOVA was used to determine associations between the polymorphisms and metabolic variables, adjusting for age, gender, and BMI, as appropriate. Logistic regression analysis was used to test for associations of polymorphisms with premature CAD under inheritance models. The most appropriate inheritance model was selected based on Akaike information criteria and was adjusted for age, gender, and BMI. Genotype frequencies did not show deviation from Hardy-Weinberg equilibrium (HWE, *P* > 0.05). Pairwise linkage disequilibrium (LD,  D′) estimations between polymorphisms and haplotype reconstruction were performed with Haploview version 4 : 1 (Broad Institute of Massachusetts Institute of Technology and Harvard University, Cambridge, MA, USA).

### 2.4. Functional Prediction Analysis

We predicted the potential effect of the* PLA2G5* SNPs using the TFSearch program (http://www.cbrc.jp/research/db/TFSEARCH.html).

## 3. Results

General characteristics of the population studied are shown in Tables 1 and 2.

### 3.1. Association of Polymorphisms with Premature CAD

Observed and expected frequencies in the polymorphic sites were in HWE. Similar distribution of the rs11573185 and rs2148911 polymorphisms was observed in both groups. Under dominant, recessive, and additive models adjusting for age, gender, and BMI, the rs11573191 polymorphism was associated with increased risk of premature CAD as compared to controls (OR = 1.51, 95% CI: 1.14–1.99, *P*_dom⁡_ = 3.5 × 10^−3^; OR = 2.95, 95% CI: 1.12–3.76, *P*_rec_ = 0.023; OR = 1.51, 95% CI: 1.17–1.94, *P*_add_ = 1.2 × 10^−3^) (Table 3). The statistical power estimated with QUANTO software (http://hydra.usc.edu/GxE/) to detect an association between premature CAD and controls was 0.88 for rs11573191.

### 3.2. Association of the Polymorphisms with Metabolic Parameters and Cardiovascular Risk Factors

The effect of the three polymorphisms on various metabolic parameters and cardiovascular risk factors was analyzed in premature CAD patients and controls. No associations were observed in this analysis (data is not shown).

### 3.3. Haplotype Analysis and Functional Effect

The three* PLA2G5* polymorphisms were in strong linkage disequilibrium (*D*′ > 0.95) and four haplotypes were observed:* AGG, CGG, CAG, *and* CGA.* The* CGA *haplotype was associated with increased risk of premature CAD (OR = 1.49, 95% CI: 1.15–1.93, and* P* = 0.0023) (Table 4). The effect of the haplotypes on diverse metabolic parameters and cardiovascular risk factors was analyzed in premature CAD patients and healthy controls. Only the* CGA* haplotype was associated with increased risk of hypertension in the group of patients with premature CAD (OR = 1.75, 95% CI: 1.17–2.60, and* P* = 0.0072) (data is not shown). This analysis was adjusted for age, gender, medication, and BMI.

Based on SNP functional prediction software, the rs11573191 polymorphism seems to be functional. This polymorphism modifies the binding affinity of the transcriptional factor MZF1, having greater affinity by the* A* allele. The differences in affinity could have important consequences in the expression of sPLA_2_-V protein.

## 4. Discussion

The role of sPLA_2_-IIA in atherogenesis has been well studied; however, the involvement of sPLA_2_-V is less understood. sPLA_2_-V is highly expressed in the heart and is present in other tissues as well, including eye, placenta, lung, and brain [17–21]. A number of human cells, including macrophages, neutrophils, bronchial and renal tubular epithelia, subendocardial cells (cardiomyocytes), and interstitial fibroblasts of gastric submucosa, have been shown to express sPLA_2_-V [20, 22–26]. Recently, Ohta et al. [27] identified a unique function of sPLA_2_-V in activation of macrophages and in their capacity to recruit T cells to amplify the effector phase of pulmonary inflammation. However, the possible effect of the sPLA_2_-V in the developing of atherosclerosis is contradictory. Enzyme deficiency in sPLA_2_-V-null mice leads to marked attenuation of airway inflammation [28, 29] and reduced atherosclerosis [9, 30]. It has been reported that sPLA_2_-V can hydrolyze phospholipids in LDL, leading to the production of proatherogenic modified LDL in vitro [7].* PLA2G5* overexpression in bone marrow cells worsens atherosclerosis, whereas its deficiency decreases modestly the atherosclerosis [9]. In the same way, the* PLA2G5* deficiency does not affect the atherosclerotic lesion development in mice [30] and pan-sPLA2 inhibitor varespladib did not reduce the risk of cardiovascular events after acute coronary syndrome [31]. The genes that encode sPLA_2_-IIA and sPLA_2_-V molecules are linked in a negative orientation on the same chromosome [14]. Polymorphisms in both genes have been associated with variations in the lipid levels [15, 32]. In the present work, three* PLA2G5* gene polymorphisms (rs11573185, rs2148911, and rs11573191) were analyzed in order to establish their role as susceptibility markers for premature CAD, metabolic parameters, and cardiovascular risk factors. The functional prediction software used here predicted that the rs11573191 polymorphism is functional with an effect on the affinity of the sequence for the MZF1 transcriptional factor. The* A* allele of this polymorphism presents major affinity for the transcription factor than the* G* allele, having important consequences on sPLA_2_-V production. This result obtained using informatics software is in agreement with our genetic results because, in the association analysis, the rs11573191* A *allele was associated with increased risk of developing premature CAD. However, our study did not include expression analysis and we have no evidence that the* PLA2G5* expression is different in premature CAD patients with the risk allele. The distribution of the other two* PLA2G5 *polymorphisms was similar in CAD patients and healthy controls. Recently, Holmes et al. [33] using data from the Advanced Study of Aortic Pathology identified that the* PLA2G5* rs525380 polymorphism was strongly associated with* PLA2G5* mRNA expression levels. However, the association of this polymorphism with sPLA activity and coronary heart disease was not corroborated. This polymorphism was not included in our analysis. Wootton et al. [15] studied seven* PLA2G5* polymorphisms in patients with type II diabetes mellitus to investigate the association of these polymorphisms with coronary heart disease risk factors. Of the seven SNPs, three of them (rs11573185, rs11573203, and rs11573248) showed significant association with cholesterol and LDL levels. In our study, none of the studied polymorphisms was associated with lipid levels in premature CAD or healthy controls. In the study by Wootton et al. [15], the haplotype analysis showed associations of some haplotypes with significantly higher cholesterol and LDL. In our work, the three studied polymorphisms were in linkage disequilibrium, and one of the haplotypes (*CGA*) was associated with risk of developing premature CAD and with hypertension in the premature CAD patients. This haplotype included the* A *allele associated independently with the disease. In a previous work, Mancini et al. [34], using a genome-wide association analysis in a spontaneously hypertensive rat model, identified four candidate genes for hypertension, one of them was the* PLA2G5* gene. This agrees with our study, in which an association of the* PLA2G5* haplotype with hypertension was detected.

Study limitations need to be addressed. This study only included the analysis of three polymorphisms of the* PLA2G5* gene. Considering that this is the first work to report an association of the* PLA2G5* polymorphisms with premature CAD and hypertension, replication in another group of patients is necessary. The predicted functional consequences of the rs11573191 polymorphism, using informatics tools, need experimental testing.

## 5. Conclusion

In summary, our study demonstrates the association of the* PLA2G5* rs11573191 polymorphism with premature CAD and with hypertension in this group of patients. According to the informatics software, this polymorphism had a functional effect in modifying the affinity of the sequence by the MZF1 transcription factor. The associations reported in the present work should be explored in other populations to establish the true role of these polymorphisms in cardiovascular diseases.

## Figures and Tables

**Table 1:** Demographic characteristics of the studied population.

	Controls	Premature CAD	*P*
	(*n* = 749)	(*n* = 707)	
Age (years)	53.99 ± 9.82	53.3 ± 7.4	0.982
Gender (% male)	49.7	81.4	<0.0001
Body mass index (kg/m^2^)	28.47 ± 4.44	28.77 ± 4.29	0.184
Obesity (%)	31.5	35.3	0.067
Waist circumference (cm)	94.38 ± 11.62	98.06 ± 11.32	<0.0001
Central obesity (%)	79.7	82	0.144
Total abdominal fat (cm^2^)	438.44 ± 163.17	424.59 ± 166.62	0.110
Subcutaneous abdominal fat (cm^2^)	181.30 ± 120.63	248.95 ± 110.14	<0.0001
Visceral abdominal fat (cm^2^)	157.09 ± 70.70	175.71 ± 81.70	<0.0001
Visceral/subcutaneous adipose tissue ratio	1.99 ± 1.00	1.54 ± 0.81	<0.0001
Current smokers (%)	21.4	12.3	<0.0001
Former smokers (%)	36.8	64.4	<0.0001
Hypertension (%)	24.9	64.9	<0.0001
Hypertensive medication (%)	11.2	89.9	<0.0001
Diastolic blood pressure (mmHg)	74.53 ± 10.09	74.16 ± 10.25	0.497
Systolic blood pressure (mmHg)	121.56 ± 18.83	121.48 ± 19.45	0.942
Heart rate (bpm)	65.54 ± 9.20	64.99 ± 11.00	0.301

Data are expressed as means ± SD; log-transformed values were used for statistical analysis.

*P* values were estimated using ANOVA for continuous variables and Pearson's Chi-square test for categorical values.

CAD: coronary artery disease.

**Table 2:** Comparison of biochemical parameters in individuals with premature coronary artery disease and controls.

	Controls	Premature CAD	*P*
	(*n* = 749)	(*n* = 707)	
Total cholesterol (mg/dL)	19338 ± 36.46	169.31 ± 48.18	<0.0001
TC > 200 mg/dL (%)	40.0	20.7	<0.0001
HDL-C (mg/dL)	46.61 ± 13.69	39.90 ± 10.82	<0.0001
Hipo-a-lipoproteinemia (%)	51.2	57.5	0.009
LDL-C (mg/dL)	118.69 ± 32.37	97.22 ± 40.25	<0.0001
Triglycerides (mg/dL)	170.47 ± 113.01	193.72 ± 127.26	<0.0001
Hypertriglyceridemia (%)	46.9	59.8	<0.0001
ApoAI (mg/dL)	139.50 ± 43.68	119.32 ± 26.74	<0.0001
ApoB (mg/dL)	91.32 ± 28.85	82.71 ± 31.01	<0.0001
Statin and/or fibrate treatment (%)	4.2	15.4	<0.0001
Type 2 diabetes mellitus (%)	7.6	35.3	<0.0001
Glucose (mg/dL)	100.75 ± 35.75	112.33 ± 44.31	0.001
HOMA-IR	5.29 ± 8.38	6.73 ± 6.00	<0.0001
Insulin (*μ*U/mL)	20.32 ± 13.82	24.23 ± 17.26	<0.0001
Metabolic syndrome (%)	46.3	46.1	0.488
Uric acid (mg/dL)	5.47 ± 1.78	6.07 ± 2.06	<0.0001
Creatinine (mg/dL)	0.84 ± 0.25	0.92 ± 0.31	<0.0001
Alanine transaminase (IU/L)	27.75 ± 12.43	28.07 ± 11.49	0.619
Aspartate transaminase (IU/L)	26.84 ± 11.34	27.97 ± 17.77	0.222
Alkaline phosphatase (IU/L)	83.07 ± 28.36	78.99 ± 25.97	0.004
Gamma-glutamyl transpeptidase (IU/L)	35.91 ± 32.83	44.36 ± 44.63	<0.0001

Data are expressed as means ± SD; log-transformed values were used for statistical analysis.

*P* values were estimated using ANOVA for continuous variables and Pearson's Chi-square test for categorical values.

CAD: coronary artery disease.

**Table 3:** Association of the rs11573185, rs2148911, and rs11573191 *PLA2G5* gene polymorphisms with premature CAD.

rs11573185	Genotype frequency (%)			MAF	Model	OR (95% CI)	*P*
	A/A	A/C	C/C				

Control (*n* = 749)	0.410	0.437	0.153	0.371			
Premature CAD (*n* = 707)	0.395	0.462	0.142	0.373	Dominant additive	1.21 (0.97–1.52) 1.10 (0.94–1.28)	0.089 0.250

rs2148911	Genotype frequency (%)			MAF	Model	OR (95% CI)	*P*
	G/G	G/A	A/A				

Control (*n* = 749)	0.780	0.206	0.015	0.117			
Premature CAD (*n* = 707)	0.782	0.203	0.014	0.115	Dominant additive	1.08 (0.83–1.41) 1.07 (0.84–1.36)	0.560 0.610

rs11573191	Genotype frequency (%)			MAF	Model	OR (95% CI)	*P*
	G/G	G/A	A/A				

Control (*n* = 749)	0.825	0.166	0.009	0.092			
Premature CAD (*n* = 707)	0.777	0.202	0.021	0.122	Dominant recessive additive	**1.51 (1.14–1.99)** **2.95 (1.12–3.76)** **1.51 (1.17–1.94)**	**3.5 × 10** ^−3^ **0.023** **1.2 × 10** ^−3^

Associations were tested using logistic regression adjusting for age, gender, and BMI.

CAD: coronary artery disease; MAF: minor allele frequency.

**Table 4:** Haplotype frequencies in premature CAD patients and healthy controls.

rs11573185	rs2148911	rs11573191	Frequencies			OR (95% CI)	*P*
			Total	Control	Premature CAD		
*A*	*G*	*G*	0.625	0.625	0.6256	1	—
*C*	*G*	*G*	0.151	0.165	0.1367	0.86 (0.69–1.08)	0.19
*C*	*A*	*G*	0.115	0.115	0.1153	1.12 (0.88–1.44)	0.36
*C*	*G*	*A*	0.105	0.091	0.1207	**1.49 (1.15–1.93)**	**0.0023**

The ORs were adjusted for age, gender, medication, and BMI.

The *AGG* haplotype was used as reference.
